# Supplementary material for: Comparison of Placental HSD17B1 Expression and Its Regulation in Various Mammalian Species
Source: Animals (Basel). 2023 Feb 10;13(4):622. doi: 10.3390/ani13040622 (PMC9951672; doi:10.3390/ani13040622)
Supplement: Supplementary file 1 [file animals-13-00622-s001.zip › animals-2135501-supplementary.pdf]

Supplementary Table S1. Primers used in each experiment.

PCR primers	Forward primers	Reverse primers
**RT-PCR**		
murine Cyp11a1	F- ttccgcttttcctttgagtccat	R- gtgtctccttgatgctggcttt
murine Hsd3b1	F- actgcaggaggtcagagct	R- gccagtaacacacagaatacc
murine Hsd3b6	F- tctggaggagatcagggtc	R- gcccgtacaaccgagaatatt
murine Cyp17a1	F- aaataataacactggggaaggc	R- tgggtgtgggtgtaatgagatgg
murine Cyp19a1	F- tcaataccaggtcctggcta	R- gtatgcactgattcacgttc
murine Hsd17b1	F- actgtgccagcaagtttgcgc	R- aagcggttcgtggagaagtag
murine Gapdh	F- accacagtccatgccatcac	R- tccaccaccctgttgctgta
guinea pig Cyp11a1	F- ttgagtccatcaccaatgtca	R- tggcctgaatgttctcatagg
guinea pig Hsd3b	F- atcgtccacttgttggtgaag	R- ctcattggtgttctggacgat
guinea pig Cyp17a1	F- tgcatgatctgcttcagtgtc	R- tgcatgatctgcttcagtgtc
guinea pig Cyp19a1	F- gcacagcagagaaattggaag	R- caccaggacgactttcatcat
guinea pig Hsd17b1	F- actgtgcaagcaagttcgcgc	R- aagcgctcggtgcagaagtag
guinea pig Gapdh	F- accacagtccatgccatcac	R- tccaccaccctgttgctgta
ovine CYP11A1	F- gtgtctcaggacttcgtcagc	R- ccatctcgtacaagtgccact
ovine HSD3B1	F- tgttggtggaggagaaggatctg	R- tgggtacctttcacattgacgttc
ovine CYP17A1	F- tcctgtcagacaaccaaaagg	R- cacaacccttcatcttttcca
ovine CYP19A1	F- gtccgaagttgtgcctattgccagc	R- cctccaacctgtccagatgcttgg
ovine HSD17B1	F- ttgtgcgagagtctggcgattct	R- aggaatcgctcggtggtgaagta
bovine CYP11A1	F- gtgtctcaggacttcgtcagc	R- ccatctcgtacaagtgccatt
bovine HSD3B1	F- tgttggtggaggagaaggatctg	R- tgggtacctttcacattgacgttc
bovine CYP17A1	F- tcctgtcagacaaccaaaagg	R- cacaacccttcatcttttcca
bovine CYP19A1	F- aagccttagaggatgatgtc	R- ggtctcgtctggatgcaagg
bovine HSD17B1	F- ttgtgcgagagtctggcgattct	R- aggaatcgctcggtggtgaagta
bovine GAPDH	F- tgttccagtatgattccaccc	R- tccaccaccctgttgctgta
human CYP11A1	F- tagtgtctccttgatgctgg	R- gaaaggaagtgttcaccacg
human HSD3B1	F- tggaatctgaaaaacggcggc	R- tgagcttgtgcccttgtcact
human HSD3B2	F- tggaatctaaaaaatggtgat	R- tgggcttgtgcccctgttgcc
human CYP17A1	F- gaaggcatcatagacaacctga	R- acattctggtcaatctcctcgt
human CYP19A1	F- ctggaagaatgtatggactt	R- gatcatttccagcatgtttt
human HSD17B1	F- atgacgtttattgcgccagc	R- ccggaacgtcgccgaacactt
human β-actin	F- gggaaatcgtgcgtgacattaag	R- tgtgttggcgtacaggtctttg
Bisulfite		
ovine HSD17B1	F- gatataataaaggggtgggga	R- ataataaataccactatac

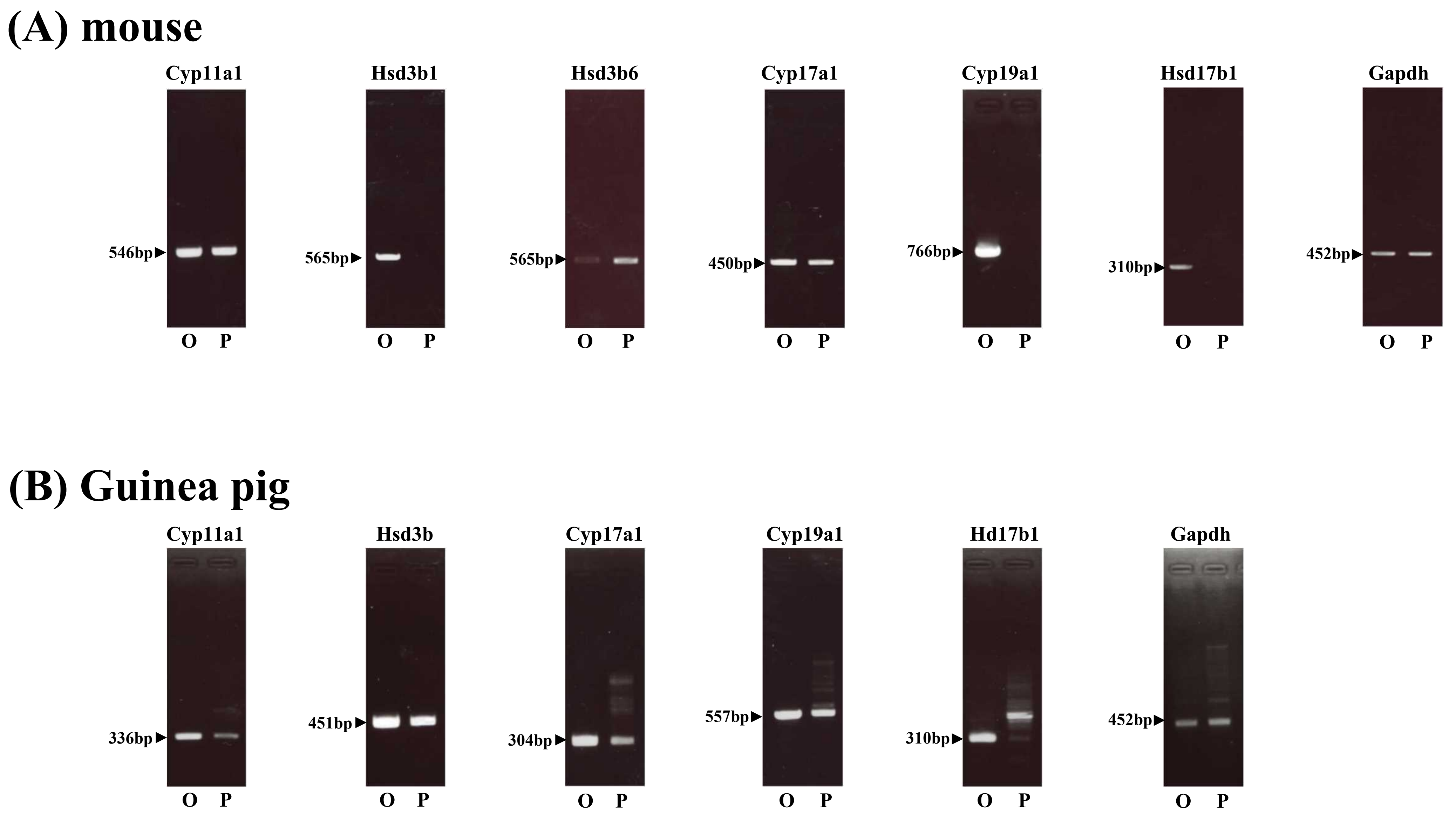

Figure S1. The whole images of RT-PCR analyses in Figure 2.
Lanes O and P represent an ovary and placenta, respectively.

(C) Porcine

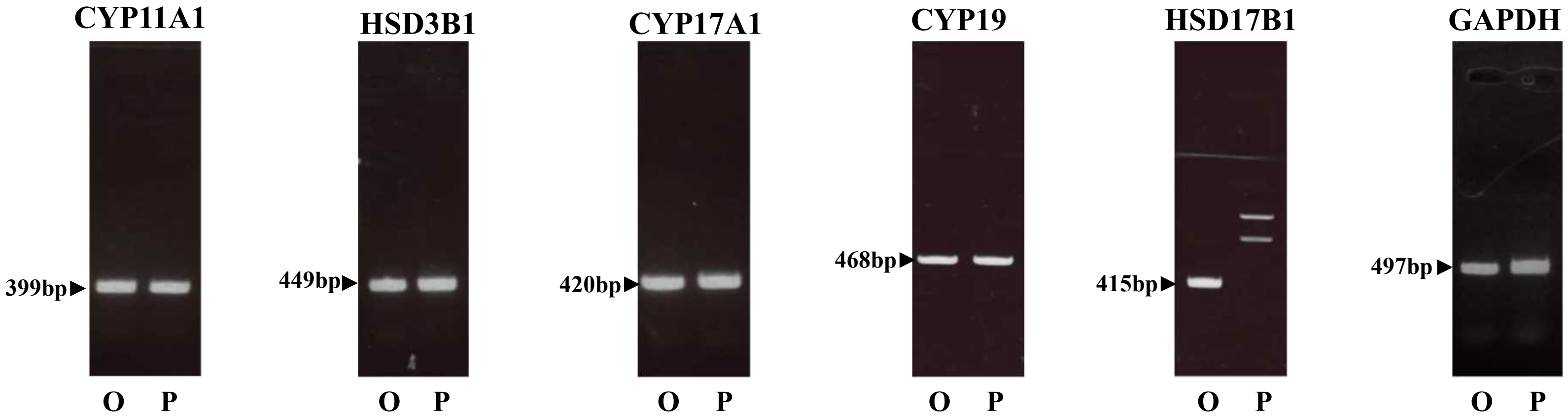

(D) Ovine

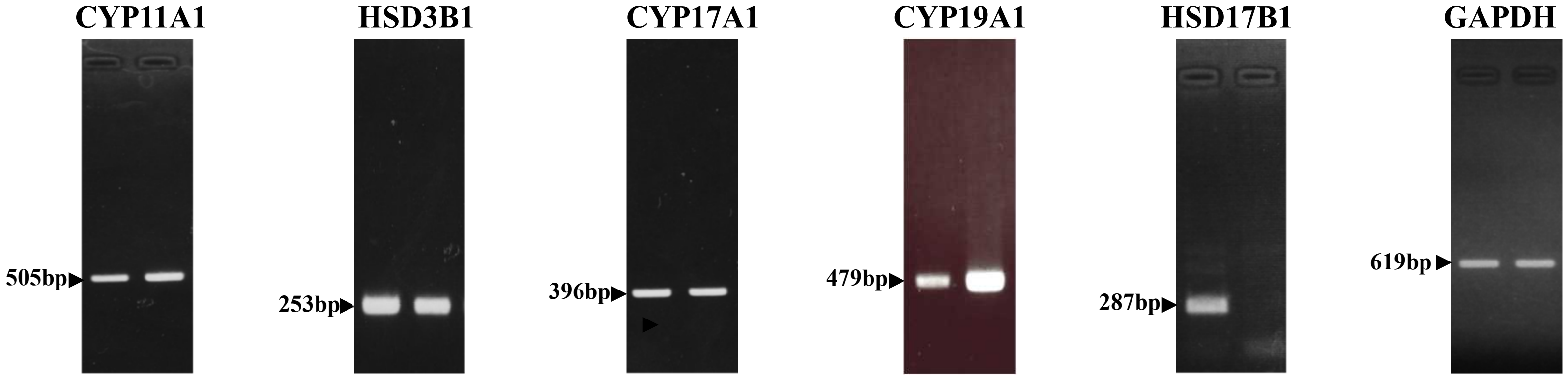

Figure S1. The whole images of RT-PCR analyses in Figure 2 (continued).
Lanes O and P represent an ovary and placenta, respectively.

(E) Bovine

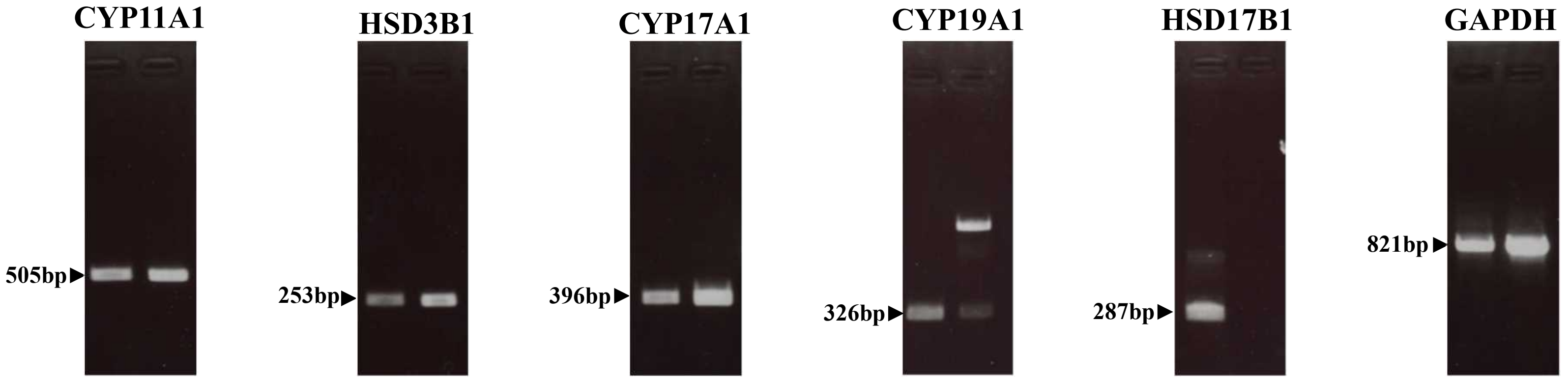

(F) human

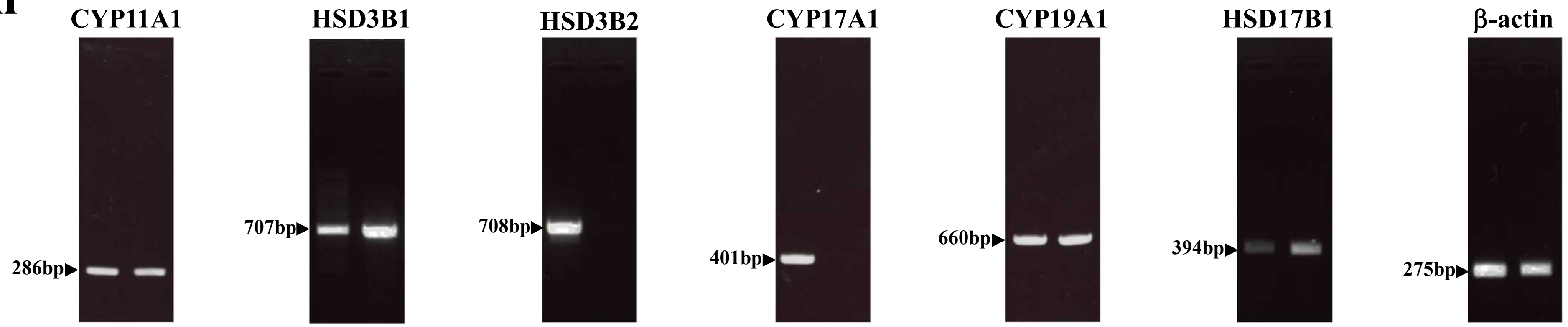

Figure S1. The whole images of RT-PCR analyses in Figure 2 (continued).
Lanes O and P represent an ovary and placenta, respectively.

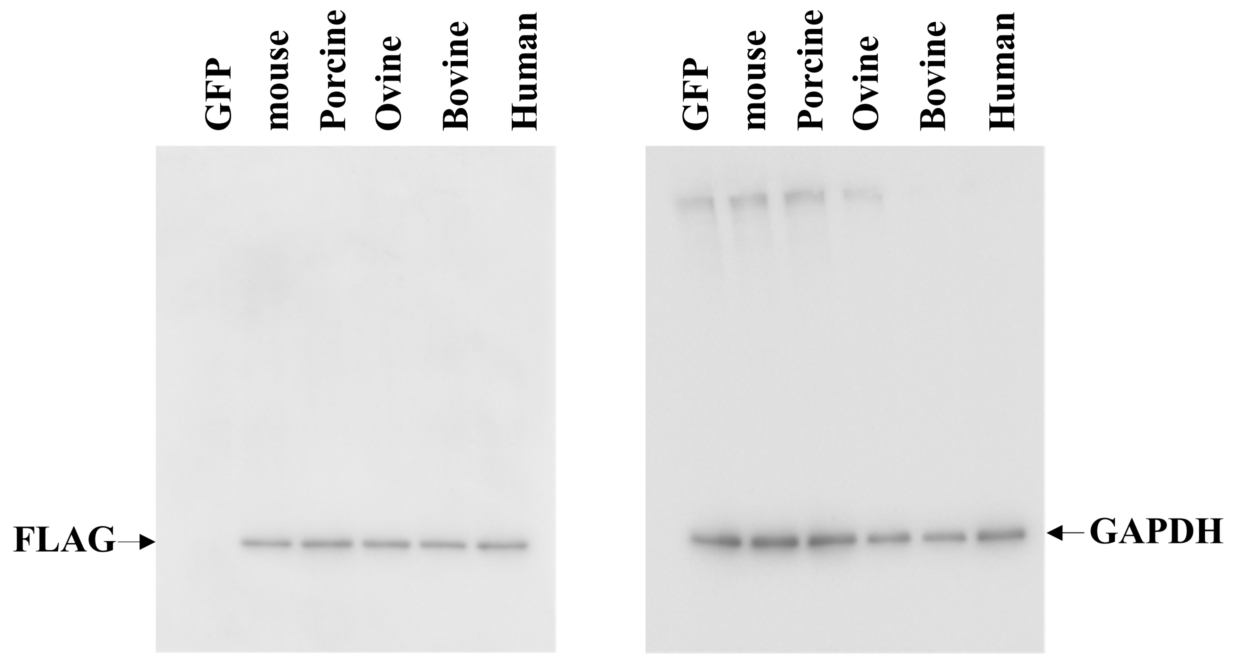

Figure S2. The whole images of western blot analyses in Figure 4(A).
